# Hemangioblast: origin of hemangioblastoma in von Hippel-Lindau (VHL) syndrome

**DOI:** 10.18632/oncoscience.447

**Published:** 2018-08-22

**Authors:** Herui Wang, Zhengping Zhuang, Chi-Chao Chan

**Affiliations:** Chi-Chao Chan: National Eye Institute, National Institutes of Health, Bethesda, MD 20892, USA

**Keywords:** von Hippel-Lindau (VHL) syndrome, retinal cap- illary hemangioblastoma (RCH), hemangioblast, tumorlet cell, mouse model

von Hippel-Lindau (VHL) disease is a highly penetrant tumor predisposition syndrome in which retinal and central nervous system (CNS) hemangioblastoma is one of the major symptoms [1]. Retinal capillary hemangioblastoma (RCH) is a retinal benign tumor that represents the first manifestation in more than 50% of VHL patients. RCH accounts for significant visual loss in affected individuals with 25% of affected globes harboring visual acuity less than 20/160 [2]. Although VHL-associated RCH is believed to be secondary to abberant upregulation of hypoxia-inducible factors caused by *VHL* mutations, the mechanisms underlying the pathophysiology of RCH remain unclear. Lack of appropriate animal models of VHL-associated RCH hinders further investigation into RCH pathophysiology and the development of novel treatment strategies [3].

One question in this field is regarding the primordial cell origin of RCH in VHL syndrome. Previous tissue- specific *Vhl* knockout mouse models have aided in the understanding of VHL syndrome development in the kidney, liver, genitourinary tract, and pancreas [3], but none of them showed RCH lesions, indicating that RCH originates from a different cell type. Deric M. Park and colleagues reported that CNS hemangioblastomas resected from VHL patients express several mesodermal markers including brachyury, stem cell leukemia (SCL) and fetal liver kinase 1 (FLK-1), which is consistent with the embryonically derived hemangioblasts [4]. These neoplastic cells can also be differentiated into multiple hematopoietic cell lineages, suggesting that VHL-associated hemangioblastomas are derived from mesoderm-derived, embryonically arrested hemangioblasts.

**Figure 1:**
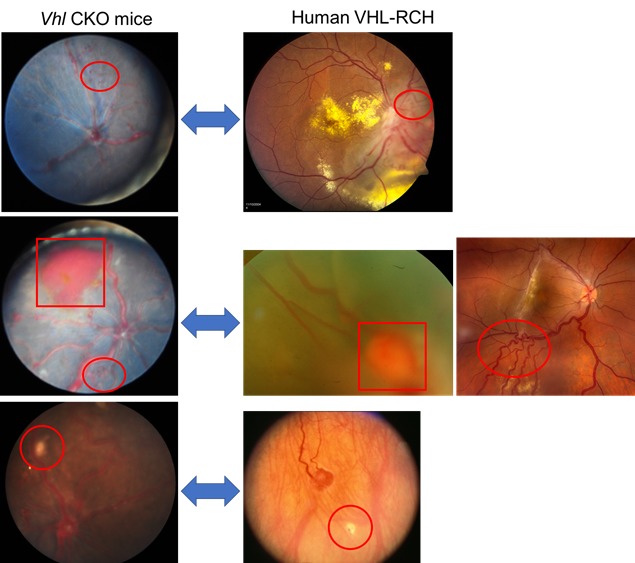
Comparison between the retinal vascular lesions in the mouse model and VHL patients Neovasculature (ovals in the top and middle panel), hemorrhage (rectangles in the middle panel), and bright dots (circles in the bottom panel) are all observed in both Vhl mouse model and VHL-RCH patients. (The three left panels are from Figure 2B, 4B and 4D of Ref. 5).

In our study, we proved the above hypothesis by conditionally knocking out *Vhl* in hemangioblast-derived cells after birth using tamoxifen-inducible *Scl*-Cre-ERT2 transgenic mice [5]. Following tamoxifen induction around two weeks after birth, more than half of the conditional knock out mice exhibited manifestations of VHL-associated RCH, including vascular defects (dilated tortuous vessel, neovasculature, hemorrhage), and retinal red or yellow grayish lesions [5]. Clinical manifestation was observed by comparing the retinal lesions with the fundus photographs taken from the mouse model and VHL patients (Figure 1). Several retinal lesions in mice mimic that in VHL patients. Histologically, SCL positive “tumorlet” cell clusters with elevated VEGF expression are observed around the dilated retinal vessels in the *Vhl* conditional knockout mice [5]. The “tumorlet” cells are reminiscent of the pre-tumor hemangioblast cell clusters observed during RCH development in VHL patients [5, 6]. These results suggest that *Vhl* mutation alone in the hemangioblast-derived cells is sufficient for the tumor initiation of VHL-associated RCH.

Despite of the high similarity between the retinal vascular lesions in our mouse models and that in the VHL patients, we also observed a phenotypic difference in the mouse model: 46% (13/28) of *Vhl* mutant mice exhibited persistent fetal vasculature (PFV) in the vitreous humor. This result indicates that loss of VHL protein in the retinal vasculature may disrupt the programmed regression of hyaloid vessels. PFV is rarely found in VHL patients, probably because the fetal hyaloid artery regression occurs during the third trimester, prior to loss of heterozygosity of *VHL* in somatic cells after birth. However, hyaloid artery completely regresses about three weeks after birth in mice, later than the tamoxifen-induced *Vhl* deletion.

Dilated tortuous vessels and retinal red, orange or grayish lesions in the affected *Vhl* mutant mice were monitored until 9 months of age. However, obvious progression of these lesions was not observed in the *Vhl* mutant mice. None of the RCH-like lesions developed into large tumors in mice. This is consistent with the fact that none of the conditional knockout mice targeting *Vhl* in other tissues developed classical tumors as shown in the VHL patients [3]. Based on the previous reports that loss of VHL protein didn’t promote tumor growth in primary cells but did lead to extremely proliferative xenografts of renal cell carcinoma (RCC) cell lines [7], we hypothesize that loss of *Vhl* gene in hemangioblast-derived cells is sufficient for the initiation of *de novo* tumorigenesis of VHL-associated hemangioblastoma, while additional mutations and/or environmental factors are needed for the tumor progression. Incorporation of other oncogenic mutations into this *Vhl* mutant model may be able to promote the tumor growth of the retinal “tumorlet” clusters.

In summary, our findings provide a phenotypic recapitulation of VHL-associated RCH in a murine model that may be useful to study RCH pathogenesis and therapeutics aimed at treating ocular VHL.
